# One-session asthma curriculum for all K–8th grade students: impact on knowledge, attitudes, and self-efficacy

**DOI:** 10.3389/falgy.2026.1667661

**Published:** 2026-04-09

**Authors:** Caroline Luff, Alexandra Knitter, Bolanle Ogbara, LaToya Gregory, Monica Kowalczyk, Anna Volerman

**Affiliations:** University of Chicago, Chicago, IL, United States

**Keywords:** all-student education, asthma education, asthma knowledge, attitudes, children, pediatric asthma, peer support, school-based health

## Abstract

**Background:**

Schools are critical places to support children with asthma. Few school-based interventions focus on classmates without asthma, who can influence peers’ asthma self-management.

**Objective:**

To describe a one-session asthma curriculum for all Kindergarten-8th grade students and evaluate impact on knowledge, attitudes, and self-efficacy.

**Methods:**

In collaboration with three Chicago schools, we adapted and implemented a one-session asthma curriculum for K-8th grades. Students completed a post-session survey to assess asthma knowledge, attitudes, and self-efficacy. Pearson's Chi-squared tests analyzed differences between grades and student-reported asthma diagnosis.

**Results:**

In total, 603 students participated in a session, of which 485 completed the survey; 19% reported having asthma. Most students correctly answered knowledge questions on pathophysiology (82%), transmission (79%), symptoms (65%), triggers (65%), medication use (90%), and episode management (51%). More older students understood pathophysiology (85% vs. 72%, *p* = 0.002), medication use (93% vs. 83%, *p* = 0.002), and episode management (55% vs. 31%, *p* < 0.001), vs. younger students. Of all students, 83% indicated positive attitudes about asthma care and 49% about physical activity participation, with differences based on grade (asthma care: older 86% vs. younger 74%, *p* = 0.002) and diagnosis (physical activity participation: asthma 63% vs. no asthma 45%, *p* = 0.002). Most students indicated positive self-efficacy to help classmates with asthma episodes (74%), with more younger students doing so than older students (83% vs. 71%, *p* = 0.01).

**Conclusion:**

Students had high asthma knowledge and self-efficacy after a one-session all-student asthma curriculum; attitudes were mixed. School-based asthma education is feasible and has positive outcomes for all K–8th grade students.

## Introduction

More than 40% of school-aged children and adolescents in the United States have at least one chronic health condition (1). In addition to the health effects, children with chronic diseases have higher rates of school absenteeism, which is associated with lower academic achievement (2). To minimize such negative outcomes, children with chronic diseases require support in and outside of clinical settings, including in schools, where children spend the majority of their days. Research also highlights schools as optimal venues to support children's health, as they can reach a large number of children, including many who rely on schools for health services due to barriers to healthcare access (3).

School-based care is critical to enable optimal engagement for the 4.3 million school-aged children with asthma (4). Asthma is a leading cause of school absenteeism, accounting for 13.8 million missed school days annually (5). Further, research shows that children with inadequate asthma control have lower quality of life, are more likely to be bullied, and less frequently attend school activities (6–8). Inadequately controlled asthma can also interfere with students’ ability to fully engage in school, which can have detrimental lifelong consequences, such as increased disability, fewer job opportunities, and limited community interactions (3). As such, schools are integral places to promote asthma care by providing disease-specific education, supporting medication adherence, and reducing environmental exposures during the school day (9).

School-based asthma interventions are shown to positively impact affected children (10). Particularly, asthma education is a valuable tool to support disease control, reducing hospital, emergency department, and clinic visits by affected children (11). However, such asthma education typically focuses on students with asthma and does not commonly include students without asthma, who directly interact with and impact the daily experience of affected students (12). For instance, studies demonstrate that children with asthma who experience greater peer victimization have lower asthma control and self-efficacy (13). Further, research indicates lower social support is significantly associated with lower medication adherence among children with asthma (14). As such, peer support can mitigate adverse outcomes for affected students.

One effective method to encourage such peer support is asthma education for all students, including both students with and without asthma (15). To date, school-based asthma education programs for all students primarily target a small number of grade levels, with various programs that collectively span 3rd–10th grades (12, 16–23). Further, these programs are delivered over multiple sessions (12, 17–21, 23), which are led by school nurses (22), teachers (12, 17, 20, 21), or peers (18, 19, 23). Evaluation of these programs demonstrates they improve asthma knowledge (12, 16–18, 20–24). Also, few programs examine attitudes, with some demonstrating improved attitudes toward students with asthma (16, 17), while others show limited effects on attitudes (18, 21, 23). When all-student asthma education is part of a more comprehensive initiative to support asthma in schools, studies show reduced symptoms among students with asthma (25) and mixed effects on quality of life and school absenteeism of affected students (22, 24).

Although asthma education for all students has expanded in the past two decades, such programs remain limited in their intended audiences, implementation, and evaluation. First, these programs primarily focus on students in 3rd grade and higher. Few such programs are tailored to younger children (K–2nd grade) (24, 25), who have less asthma knowledge and a greater chance of bullying and victimization than older students (8, 26). In fact, research shows students in higher grades have more favorable attitudes toward peers with asthma, underscoring the importance of asthma education for younger students (27). In terms of program implementation, multi-session asthma curricula are often challenging to integrate into schools, particularly in marginalized communities most affected by asthma, given limited time, resources, and staffing (28). A one-session asthma education program may be more feasible to incorporate into schools, yet there remains a paucity of published research on such programs for all students. Further, current evaluation efforts examine effects on students with asthma, overlooking youth without asthma, who have lower disease knowledge (29). Also, despite research showing disease stigma as a key barrier to school-based care for chronic illness (30, 31), few all-student programs evaluate their impact on students’ attitudes toward children with asthma, a key metric to understand potential effects of education on peer support (16–18, 21, 23). Likewise, few programs analyze effects on self-efficacy (23), a critical component of asthma care that can improve negative attitudes toward asthma and offset perceived barriers to asthma self-management (32). Understanding programs’ impact on self-efficacy is key because children with asthma who have high self-efficacy are more adherent to treatment, leading to better asthma outcomes (32).

To fill these gaps in the reach, implementation, and comprehensive evaluation of all-student asthma education, we describe the adaptation of a one-session asthma curriculum for all kindergarten to 8th grade students and evaluate its impact on knowledge, attitudes, and self-efficacy.

## Methods

This cross-sectional study was a part of a multi-component asthma intervention in three schools on Chicago's South Side that spanned Kindergarten to 8th grades. The student population is largely Black (97%) and low-income (82%) (33), and childhood asthma prevalence in this community reaches 20%–30% (34). This study was approved by the University of Chicago Institutional Review Board (IRB25-1004).

### Curriculum development

We adapted a one-session school-based asthma curriculum that was initially designed for 4th-6th grade to K–8th grade. The original asthma curriculum for all 4th–6th grade students was developed and implemented by a group of asthma educators, pediatricians, community leaders, school nurses, and parents/guardians in 2017. The curriculum was designed based on asthma diagnosis and management guidelines (35), literature review of prior curricula (12, 18, 23, 25, 36), and state learning standards for physical development and health (37). It was created to support engagement of both children with and without asthma (18, 25, 38).

When adapting for K-8th grade students, the all-student asthma curriculum was revised to be relevant to all elementary school ages, with changes made based on findings from the evaluation of the original curriculum alongside literature review of chronic illness education methods. Revisions to the curriculum's structure and content were informed by current literature on child knowledge retention and engagement (i.e., mnemonics, interactive games) (39–43) and effective school-based health intervention methods (i.e., skits, videos) (44–49) to engage the broader student population. Specific consideration was given to ensure content was age and developmentally appropriate. For instance, to adjust content for younger students (K–2nd), we incorporated instructional videos focused on basic asthma principles, whereas for older children, a greater emphasis was placed on communication and problem-solving through student-led interactive scenarios. Altogether, the adapted curriculum was designed as one 45-minute session. It included optional activities to allow flexibility within available class time (30–60 min). The curriculum's overall goal was to enhance understanding of asthma among children who do not have asthma and self-efficacy of all students; curriculum objectives and content were the same across the three schools and are listed in Table 1.

**Table 1:** Objectives and content of one-session asthma curriculum in school for all students in kindergarten-8th grade.

Objective	Key Points	Activities (with adaptations for K-2nd grade noted)
Describe asthma and how it affects the lungs/respiratory system	• Describe parts and function of respiratory system • Discuss the movement of air into the lungs • Describe how airways are affected during asthma episode (3 S: Squeezing, Swelling, Snot)	• Handout with respiratory system fill-in-the-blank: children write in name parts of respiratory system during educator-led discussion • Lung model and handout: educator compares normal airway and airway affected by asthma • Game (time permitting): Teams of 4 relay race to put together paper respiratory system pieces on the board **K–2 Adaptation:** • Handout: Color-by-number of respiratory system • No game • 3-D video of how asthma works
Describe asthma signs/ symptoms and triggers	• Describe signs, symptoms, and triggers of asthma • Discuss that asthma presents differently in each person • Describe scenarios and strategies to avoid or remove triggers	• Handouts with asthma signs, symptoms, and triggers: educator reviews asthma signs, symptoms, and triggers using • Discussion: recite triggers pneumonic together • Scenario: educator and children discuss a scenario where a child wants to go see her friend's dog but knows dogs are trigger for her asthma symptoms **K–2 Adaptation** • No scenario discussion • Video of asthma signs, symptoms, and triggers
Describe asthma medications and care	• Discuss that when asthma symptoms start, children should move away from their trigger and get an adult • Discuss the use of inhalers to treat symptoms (with spacer as relevant) • Describe quick-acting medication for asthma episodes • Discuss every child's asthma is different and that they can still be active with asthma	• Handout with inhaler pictures: educator explains various types of inhalers used for asthma • Handout with inhaler and spacer pictures: educator and children discuss importance of using inhaler and spacer • Demonstration: educator demonstrates proper inhaler and spacer use **K–2 Adaptation** • Video showing proper inhaler and spacer use
Describe how asthma feels	• Simulate difficult breathing associated with asthma	• Straw activity: educator demonstrates normal breathing and compare to difficult breathing (by pinching center of straw). Students follow the activity accordingly. **K–2 Adaptation** • No changes from older students’ program
Develop knowledge and self-efficacy to identify and effectively respond to an asthma episode	• Discuss step-by-step how to help a peer during an asthma episode • Brainstorm potential solutions for an asthma scenario to highlight the optimal actions student could take	• Scenario: educator and children discuss a scenario where a child in gym class begins to have difficulty breathing • Skit creation (time permitting): act out roles in scenario to simulate supporting a classmate with asthma • Discussion: recite acronym of what to do when a classmate is having an asthma episode **K–2 Adaptation** • No scenario discussion or skit activity • Review game
Foster empathy and understanding among children who do not have asthma	• Discuss day-to-day impact of asthma • Encourage students to support their peers with asthma to avoid triggers, take medicine properly, and tell an adult when have symptoms • Encourage students to not treat their classmates with asthma any differently	• Scenario: educator and children discuss scenario where a child does not want to take quick-relief inhaler because they believe they will be teased **K–2 Adaptation** • No scenario discussion • Video of peer-to-peer asthma support

### Curriculum implementation

The curriculum was implemented in the three schools in April/May 2024. The curriculum was taught to K–8th grade students in physical education and science classes. The sessions were led by a community health worker who had training and experience in asthma, as well as working with youth and families.

### Evaluation

Immediately post-session, students anonymously completed a survey on asthma knowledge, attitudes, and self-efficacy [version 1] (Supplementary Appendix A–C). The survey consisted of 15 questions on: participant characteristics; asthma knowledge, attitudes, self-efficacy; and curricular impact. These survey items were adapted from previously validated tools and tailored to age-appropriate literacy levels (31, 50). Asthma knowledge was assessed with true/false and multiple-choice questions for the six curricular topics (pathophysiology, transmission, symptoms, triggers, medication use, and episode management). Asthma attitudes, self-efficacy, and impact were evaluated with Likert-scale questions (three-point: agree/neutral/disagree). The two attitudes questions focused on the ability of children with asthma to participate in physical activity and the cause of asthma episodes. The self-efficacy question centered on students’ preparedness to help a classmate in the event of an asthma episode. The curricular impact questions asked about the students’ understanding of asthma and their likelihood of helping a classmate with asthma because of the session. Questions about characteristics included gender, grade level, and student-reported asthma diagnosis.

After implementing the curriculum in the first two schools, the team revised the survey based on feedback from the community health worker that younger students experienced difficulty answering the questions. A modified version of the post-survey [version 2] was created for younger age groups (K–2nd) alongside a revised version for older students (3rd–8th), which covered the same topic areas while incorporating simplified language and supplementary visual representations for response options.

### Data analyses

Descriptive statistics were generated for all questions. Each of the six knowledge questions was scored as correct or incorrect. For attitudes, self-efficacy, and impact, responses were dichotomized as agree vs. neutral/disagree. Missing responses were excluded from analysis. Pearson's Chi-squared tests assessed differences between grades (younger: K–2 vs. older: 3–8) and asthma status (student-reported diagnosis: yes vs. no/unsure). For asthma status, a sensitivity analysis was conducted for yes vs. no with unsure excluded; the findings were unchanged. Significance was defined by two-tailed *p*-value of less than 0.05. Given multiple comparisons, a Bonferroni correction was performed, and we tested each individual hypothesis at alpha=0.0045. Analysis was conducted using RStudio (version 4.4.1).

## Results

In total, 603 K–8th grade students participated in a 45-minute education session, with a total of 28 sessions in April/May 2024. We received post-session surveys from 485 students (version 1: *n* = 335, version 2: *n* = 150) (Table 2). Participants were 50% male. Student-reported asthma prevalence was 19% (*n* = 93/485), which aligns with prior studies in these schools (51); of note, 16% of students indicated they were unsure if they had asthma. Table 3 shows results for asthma knowledge, attitudes, and self-efficacy for all participants and by subgroups based on grade and asthma diagnosis.

**Table 2:** Characteristics of students participating in one-session asthma curriculum in school, 2024 (*n* = 485).

Characteristics		% (*n*/total respondents)
Gender	Male	50% (231/460)
	Female	49% (227/460)
	Nonbinary	0.4% (2/460)
Grade	K–2nd	27% (126/470)
	3–8th	73% (344/470)
Asthma diagnosis (student reported)	Asthma	19% (93/485)
	No asthma	64% (312/485)
	Unsure	16% (80/485)

**Table 3:** Knowledge, attitudes, and self-efficacy of students after participating in one-session asthma curriculum in school, overall and by subgroups (*N* = 485).

Domain		Overall % (n/total respondents)	K–2nd/Younger (*N* = 126) % (n/total)	3rd–8th/Older (*N* = 344) % (n/total)	Effect size	*p*-value^a^	Asthma (*N* = 93) % (n/total)	No Asthma/Unsure^b^ (*N* = 392) % (n/total)	Effect size	*p*-value^a^
Knowledge by topic area,^c^ correct	Pathophysiology	82% (383/468)	72% (89/123)	85% (283/333)	0.14	**0**.**002**	79% (72/91)	82% (311/377)	0.03	0.5
	Transmission	79% (363/459)	74% (87/118)	80% (264/329)	0.06	0.1	77% (71/92)	80% (292/367)	0.02	0.6
	Symptoms	65% (280/431)	63% (51/81)	65% (221/339)	0.01	0.7	60% (46/77)	66% (234/354)	0.04	0.3
	Triggers	65% (276/424)	56% (42/75)	67% (228/338)	0.09	0.06	61% (46/76)	66% (230/348)	0.04	0.4
	Medication use	90% (414/460)	83% (96/116)	93% (308/332)	0.14	**0**.**002**	88% (80/91)	91% (334/369)	0.03	0.5
	Episode management	51% (202/394)	31% (17/55)	55% (182/330)	0.16	**<0**.**001**	53% (37/70)	51% (165/324)	0.01	0.8
Attitudes, positive	Physical activity and having asthma	49% (241/466)	52% (60/116)	48% (163/338)	0.03	0.5	63% (55/87)	45% (170/379)	0.14	**0**.**002**
	Cause of asthma episodes	83% (379/456)	74% (81/110)	86% (290/336)	0.14	**0**.**002**	81% (72/89)	84% (307/367)	0.02	0.5
Self-efficacy, agreement	Prepared to help classmate with asthma episode	74% (334/451)	83% (91/109)	71% (238/334)	0.11	**0**.**01**	73% (65/89)	74% (269/362)	0.01	0.8
Curriculum impact, agreement	More likely to help classmate with asthma because of session	77% (334/436)	82% (79/96)	75% (248/332)	0.07	0.1	80% (69/86)	76% (265/350)	0.04	0.4
	Understand asthma better because of session	81% (353/437)	85% (80/94)	80% (267/335)	0.05	0.2	87% (75/86)	79% (278/351)	0.07	0.09

^a^ Pearson's Chi-squared test. Significance defined as *p* < 0.05, adjusted to 0.0045 with Bonferroni correction for multiple comparisons. *P*-values are noted in bold if they are statistically significant.

^b^ For asthma status, a sensitivity analysis was conducted for yes vs. no with unsure excluded; the findings were unchanged.

^c^ For multiple choice questions that indicated to check all that apply, responses were marked correct if all correct options were selected.

### Knowledge

Among all students, a majority correctly answered knowledge questions on pathophysiology (82%), transmission (79%), symptoms (65%), triggers (65%), medication use (90%), and episode management (51%). In the analysis by grade, a greater proportion of older students correctly answered questions about pathophysiology (85% vs. 72%, *p* = 0.002), medication use (93% vs. 83%, *p* = 0.002), and episode management (55% vs. 31%, *p* < 0.001) than younger students. There were no significant differences in older vs. younger students correctly answering questions about transmission (80% vs. 74%, *p* = 0.1) and symptoms (65% vs. 63%, *p* = 0.7). When analyzing by asthma diagnosis, there were no significant differences in students’ knowledge about the six curricular topics.

### Attitudes

Student attitudes were mixed, with 49% indicating positive attitudes about children with asthma participating in physical activity and 83% doing the same about the cause of asthma episodes. There were no significant differences between older vs. younger students who reported positive attitudes about participating in physical activity (48% vs. 52%, *p* = 0.5). Meanwhile, more older students reported positive attitudes about the cause of asthma episodes than younger ones (86% vs. 74%, *p* = 0.002). In analyses by asthma diagnosis, a greater proportion of students with asthma reported positive attitudes about participating in physical activity than students without asthma (63% vs. 45%, *p* = 0.002), while there were no significant differences in attitudes about the cause of asthma episodes (81% vs. 84%, *p* = 0.5).

### Self-efficacy

Across all students, a majority indicated positive self-efficacy to help a classmate with an asthma episode (74%). More younger students indicated positive self-efficacy than older students (83% vs. 71%, *p* = 0.01; not significant based on Bonferroni correction). In contrast, there were no significant differences in students with asthma and without asthma reporting positive self-efficacy (73% vs. 74%, *p* = 0.8).

### Curriculum impact

Post-curriculum, a majority of all students indicated they understand asthma better (81%) and are more likely to help a classmate with asthma (76%) because of the curriculum. There were no significant differences in curriculum impact by grade or asthma diagnosis.

## Discussion

This study is the first, to our knowledge, to evaluate a novel one-session asthma education program for K–8th grade students in urban schools, demonstrating positive outcomes post-curriculum in terms of students’ asthma knowledge and self-efficacy, despite mixed impact on attitudes. This study demonstrates such an intervention is feasible and beneficial for schools to promote asthma awareness and can influence school culture around chronic diseases.

In our study, most students answered asthma knowledge questions in each topic area correctly post-curriculum. This high level of asthma knowledge post-curriculum aligns with literature on asthma education programs developed for similar settings, including for student populations in grades 1–10 (12, 16–20, 22, 24, 25), who are predominately Black (12, 24), and with high asthma prevalence (17, 18, 20). All of these studies involved programs with multiple educational sessions (12, 17–21, 24, 25), while our study involved one session; thus, we demonstrate it is feasible to impact knowledge with a one-session asthma education program. Notably, in our study, knowledge varied by topic area with fewer students answering correctly in episode management and triggers. These findings suggest opportunities to further revise content to optimize student knowledge. Such curriculum refinement is important as high asthma knowledge can be beneficial in building a positive school culture around asthma, with literature providing compelling evidence that health education promotes healthy behaviors among youth and open communication about their condition with peers and parents (47, 52).

When examining asthma knowledge by subgroups, students with and without asthma had similar knowledge across all topic areas, while older and younger students had similar understanding of the transmission of asthma, its symptoms, and triggers. These findings underscore our curriculum's ability to effectively engage all students, regardless of age or asthma diagnosis. However, more older students demonstrated understanding of pathophysiology, medication use, and episode management than younger students, which may result from differences in baseline knowledge, medication familiarity, and curriculum content/delivery. For instance, research indicates that older adolescents with asthma are more knowledgeable about asthma than younger adolescents (26, 29). While our study did not focus solely on students with asthma, these age-related differences in asthma knowledge may translate to all students. Similarly, the discrepancies in knowledge about medication use may reflect that younger students are less familiar with medications, due to fewer encounters through personal experience or observing family members (53). Further, research highlights most healthy children under eight years do not understand how medications work (54). Hence, these results suggest the need to revise content to optimally engage younger students, including as related to asthma medication use. The curriculum should be adapted to better account for different educational strategies needed as children develop skills in self-management, for example by incorporating more interactive play as well as cooperative learning for younger students, which are the most effective methods in chronic disease management education interventions for this age (47).

In addition to knowledge, our study revealed mixed outcomes for attitudes. Our findings mirror prior all-student education programs that report limited impact on students’ attitudes (18, 21, 23). However, our findings differ from two prior studies that show positive effects of asthma education on students’ attitudes (16, 17), a discrepancy that may be explained because they differed from ours in regard to curriculum design (i.e., booklet, multi-lesson program) and study population [i.e., older students in 6–8th grade, predominantly white (17) or high socioeconomic status (16)]. Various factors may explain the limited impact we observed, for instance, a ceiling effect, as literature suggests students without asthma generally hold positive attitudes toward students with asthma (16, 23, 29, 31, 55). Further, the 3-point Likert scale used may have limited the ability to differentiate attitudes, whereas similar all-student asthma education programs use a 4-locus attitudes scale, which is more comprehensive but may impact understanding by younger students (21, 31). Finally, the mixed attitudes about asthma we observed were surprising given the high asthma knowledge in our study population, as studies show positive correlation between asthma knowledge and asthma attitudes (29, 55).

Regarding asthma attitudes by age group, older and younger students reported similar attitudes about children with asthma participating in physical activity. However, more older students reported positive attitudes about the cause of asthma episodes, which may reflect research that outlines older students being more accepting of classmates with asthma than younger ones (27, 29). When analyzing attitudes by asthma diagnosis, we found a greater proportion of students with asthma reported positive attitudes about participating in physical activity than students without asthma, while there were no differences in attitudes about the cause of asthma episodes. Our results echo prior studies assessing students’ attitudes toward asthma, including one study that reported students without asthma had less tolerance toward children with asthma and their limitations on activity as compared to children with asthma (29). Given these findings, future curriculum refinements, such as peer teaching, role-playing, and repeated asthma education, may have greater potential to impact asthma attitudes of all students.

In contrast to mixed attitudes, our study demonstrated that post-curriculum, a majority of all students had positive self-efficacy about helping a classmate with an asthma episode. This finding was unanticipated in light of the results about attitudes, given prior research highlights higher self-efficacy is associated with more positive attitudes about asthma (56). Such research underscores that the mixed attitudes we observed could be a consequence of our evaluation methods rather than the content of the curriculum. Taken together with asthma knowledge, the students’ positive self-efficacy emphasizes the potential of the curriculum to encourage peer support for students with asthma, similar to other research revealing that asthma education increases peer support (15). Hence, our curriculum can help schools cultivate a supportive environment for students with asthma and even serve as a model for schools to incorporate education for other common chronic illnesses at school, such as food allergies. Notably, we must acknowledge that developing self-efficacy is an ongoing process requiring support from many individuals, including students’ families, healthcare professionals, teachers, and school nurses (57). For this reason, in addition to our curriculum, schools must offer regular opportunities for children to discuss and apply these concepts, as well as promote collaboration from all groups involved with asthma care (i.e., caregivers, healthcare professionals, peers).

In evaluating self-efficacy by subgroups, we found that a greater proportion of younger students indicated positive self-efficacy than older students. This result was unexpected considering that children develop increasing autonomy for asthma self-management as they mature (26). Our findings may differ because younger children in our study may have been overly confident, as suggested by literature that highlights children are more likely to report self-efficacy than objective measures of preparedness (58). Nevertheless, our curriculum shows early asthma education can support all students in feeling confident to help a classmate with an asthma episode. Further, in analyzing by asthma diagnosis, there were no differences in self-efficacy to help a classmate in the event of an episode, reinforcing the program's value to all students and potential to cultivate supportive school environments for students with asthma. While few prior all-student education programs measure effects on self-efficacy, one study that included students with asthma documented significant improvements in mean self-efficacy post-intervention (23). Along with these findings, our study emphasizes the importance of all-student education programs to promote increased self-efficacy among children, with the goal of supporting better asthma care in schools.

Following the curriculum, majority of all students indicated that they are more likely to help a classmate with asthma and understand asthma better because of the curriculum. There were no differences by grade or asthma diagnosis. As such, this result emphasizes the ability of our curriculum to be broadly accessible across all developmental stages, regardless of asthma diagnosis. Further, we provide evidence that school-based education can be an effective method to promote chronic disease awareness among school-aged children and should be considered for broader school or district-wide implementation.

For limitations, participants only completed surveys post-curriculum, and this study did not have a control group, limiting our ability to attribute asthma knowledge, attitudes, and self-efficacy directly to the curriculum. Similarly, not all participating students completed the post-survey. Also, surveys were distributed immediately after the session and did not assess medium or long-term effects. Despite these limitations, our study design represents real-world application that accounts for the pragmatic considerations of implementation in school classrooms. While the curriculum objectives and content were the same across the three schools, the survey underwent revisions in mid-stages of implementation (i.e., only one school received the revised survey) to refine questions for the students’ literacy level in K–2nd grade students. Thus, the survey tool and changes mid-implementation may have limited our ability to fully capture the effects of the curriculum on younger students. Further, the survey tool had a limited number of questions in each domain, particularly attitudes and self-efficacy, to support its completion within the timeframe in the classroom and also dichotomized the results. These measurement-related factors may have introduced biases, impacted internal validity, and limited the ability to detect meaningful changes or differences between subgroups. Lastly, our study was conducted in a high-prevalence, urban community, potentially limiting its generalizability to other populations.

Our one-session asthma curriculum was delivered to all K-8th grade students in three schools, and participating students had high asthma knowledge and self-efficacy post-curriculum; attitudes about asthma were mixed. Future research should examine pre and post-session asthma knowledge, attitudes, and self-efficacy as well as use a control group or a delayed intervention group to understand changes due to the curriculum, alongside individual-level effects. In addition, future work should evaluate the curriculum's longitudinal impact on knowledge, attitudes, and self-efficacy, as well as the impact of recurrent asthma education for all students (e.g., annual curriculum implementation) and its effect on self-management, quality of life, asthma control, and absenteeism among students with asthma. Our curriculum suggests school-based asthma education is feasible and beneficial for all K–8th grade students in classrooms, underscoring that such educational programs may promote inclusive environments for chronic disease management.

## Data Availability

The raw data supporting the conclusions of this article will be made available by the authors, without undue reservation.
