# Supplementary material for: One-session asthma curriculum for all K–8th grade students: impact on knowledge, attitudes, and self-efficacy
Source: Front Allergy. 2026 Apr 9;7:1667661. doi: 10.3389/falgy.2026.1667661 (PMC13102842; doi:10.3389/falgy.2026.1667661)
Supplement: Supplementary file 1 [file Datasheet1.pdf]

Appendix A: Survey K–8th Grade– Version 1

School Asthma Education Session

Tell us about yourself. Circle ONE option for each question.

1. School: [Name1] [Name2] [Name3]

2. Grade: K 1 2 3 4 5 6 7 8

3. Gender: Male Female Non-binary

4. Asthma: Yes, I have asthma No, I do not have asthma

Read each sentence and circle if it is true or false, or if you are unsure.

Sentence	*Circle one for each sentence*		
5. An asthma episode happens because of swelling in the airways of the lung.	True	False	Unsure
6. I can catch asthma from another person.	True	False	Unsure
7. When someone has an asthma episode, he/she should use a quick-relief inhaler (or pump).	True	False	Unsure

*For each question, circle **ONE** correct answer.*

8. What are the signs of an asthma episode?
   A. Coughing
   B. Trouble breathing
   C. Wheezing
   D. All of the above

9. The following things may trigger an asthma episode **EXCEPT**…
   A. Weather
   B. Hot foods
   C. Dust
   D. Pets

10. What can you do to support a classmate with asthma?
   A. Remind him/her to avoid triggers
   B. Laugh if he/she has to use his/her medication
   C. Do not include him/her in sports activities
   D. Tell him/her to lie down during an asthma episode

Tell us if you agree or disagree with each sentence.

Sentence	*Circle one for each sentence*		
11. Children with asthma cannot go to gym class and cannot play sports well.	Disagree	Neutral	Agree
12. When a person with asthma has an episode, it is their own fault.	Disagree	Neutral	Agree
13. I feel prepared to help a classmate or friend who is having an asthma episode.	Disagree	Neutral	Agree
14. I am more likely to help a classmate with asthma because of this session.	Disagree	Neutral	Agree
15. I understand more about asthma because of this session.	Disagree	Neutral	Agree

Appendix B: Survey K–2nd Grade– Version 2

School Asthma Education Session

Tell us about yourself. Circle ONE option for each question.

1. Grade: K 1 2 3 4 5 6 7 8

2. Gender: Boy Girl Non-binary

3. Asthma: Yes, I have asthma No, I do not have asthma I don't know

Read each sentence and circle yes, no, or maybe.

4. An asthma episode happens because of the squeeze, swell, and snot in the airways of the lung. 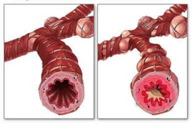	Yes No Maybe
6. I can catch asthma from another person.	Yes No Maybe
7. When someone has an asthma episode, he/she should use an inhaler (or pump).	Yes No Maybe

8. Circle one answer: what are the signs of an asthma episode?

A. Coughing 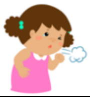	B. Trouble breathing 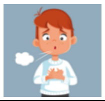	C. Wheezing/whistling 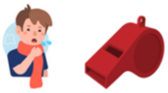	D. All of the above

9. Circle the thing that would **NOT** trigger an asthma episode.

A. Weather 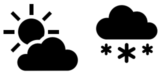	B. Hot foods 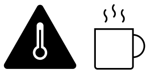	C. Dust 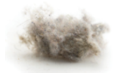	D. Pets 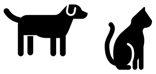

10. Circle one answer: what can you do to support a classmate with asthma?

A. Remind your classmate to move away from asthma triggers	B. Laugh if your classmate has to use their medication	C. Do not allow your classmate to play sports with you	D. Tell your classmate to lie down during an asthma episode

Tell us if you agree or disagree with each sentence.

Sentence	*Circle one for each sentence*		
11. Children with asthma cannot go to gym class and cannot play sports well.	Disagree ☹	Neither 😐	Agree ☺
12. When a person with asthma has an episode, it is their fault	Disagree ☹	Neither 😐	Agree ☺
13. I feel prepared to help a classmate or friend who is having an asthma episode.	Disagree ☹	Neither 😐	Agree ☺
14. I am more likely to help a classmate with asthma because of this session.	Disagree ☹	Neither 😐	Agree ☺
15. I understand more about asthma because of this session.	Disagree ☹	Neither 😐	Agree ☺

Appendix C: Survey 3–8th Grade– Version 2

School Asthma Education Session

Tell us about yourself. Circle ONE option for each question.

1. School: [Name1] [Name2] [Name3]

2. Grade: K 1 2 3 4 5 6 7 8

3. Gender: Boy Girl Non-binary

4. Asthma: Yes, I have asthma No, I do not have asthma I don't know

Read each sentence and circle true, false, or unsure.

Sentence	*Circle one for each sentence*		
5. An asthma episode happens because of the squeeze, swell, and snot in the airways of the lung.	True	False	Unsure
6. I can catch asthma from another person.	True	False	Unsure
7. When someone has an asthma episode, he/she should use an inhaler (or pump).	True	False	Unsure

*Circle **ONE** answer.*

8. What are the signs of an asthma episode?
   A. Coughing
   B. Trouble breathing
   C. Wheezing
   D. All of the above

9. Circle the thing that would NOT trigger an asthma episode.
   A. Weather
   B. Hot foods
   C. Dust
   D. Pets

10. Circle one answer: what can you do to support a classmate with asthma?
   A. Remind your classmate to move away from asthma triggers
   B. Laugh if your classmate has to use their medication
   C. Do not allow your classmate to play sports with you
   D. Tell your classmate to lie down during an asthma episode

Tell us if you agree or disagree with each sentence.

Sentence	*Circle one for each sentence*		
11. Children with asthma cannot go to gym class and cannot play sports well.	Disagree	Neutral	Agree
12. When a person with asthma has an episode, it is their fault.	Disagree	Neutral	Agree
13. I feel prepared to help a classmate or friend who is having an asthma episode.	Disagree	Neutral	Agree
14. I am more likely to help a classmate with asthma because of this session.	Disagree	Neutral	Agree
15. I understand more about asthma because of this session.	Disagree	Neutral	Agree
